# An evaluation of telehealth services at New York City tuberculosis clinics throughout the COVID-19 pandemic

**DOI:** 10.1371/journal.pdig.0000898

**Published:** 2025-06-24

**Authors:** Grace E. Gao, Alice V. Easton, Marco M. Salerno, Matthew Angulo, Claudia Buchanan, Deandra J. Ingram, Erica Humphrey, Marci Whitehead, Errol Robinson, Christine Chuck, Joseph Burzynski, Felicia Dworkin, Diana Nilsen, Michelle Macaraig

**Affiliations:** 1 Bureau of Tuberculosis Control, New York City Department of Health and Mental Hygiene, Queens, New York, United States of America; 2 Bureau of Public Health Clinics, New York City Department of Health and Mental Hygiene, Queens, New York, United States of America; 3 Division of Tuberculosis Elimination, Centers for Disease Control and Prevention, Atlanta, Georgia, United States of America; Iran University of Medical Sciences, IRAN, ISLAMIC REPUBLIC OF

## Abstract

In March 2020, three New York City (NYC) Department of Health and Mental Hygiene Tuberculosis (TB) clinics suspended most in-person services due to the COVID-19 pandemic and rapidly implemented telehealth to provide remote TB care. We conducted a prospective cohort study of patients with TB or latent TB infection (LTBI), who received treatment from TB clinics between April 2020 and December 2022, to compare telehealth and in-clinic services. To evaluate the success and breadth of the telehealth program, we compared patients who utilized telehealth with those who did not, analyzing differences in demographic characteristics and key outcomes, including utilization of telehealth, appointment completion, and treatment completion. “Telehealth patients” completed at least one scheduled telehealth visit during the study period. We conducted bivariate analyses comparing telehealth versus in-clinic patients. 56% (497/885) of patients with TB and 45% (954/2127) of patients with LTBI had a telehealth visit. Among patients with TB, no disparities in proportions of telehealth and in-clinic patients were observed for age (p = 0.31) or primary language spoken (p = 0.37). Among patients with LTBI, younger patients were more likely to use telehealth (p < 0.001). Using mixed-effects logistic regression models, the AOR of completing a telehealth visit was lower compared to in-clinic for patients with TB (0.77, CI:0.65-0.91). However, excluding April to June 2020, the AORs of completing a telehealth visit were comparable to an in-clinic visit for patients with TB (0.94, CI:0.77-1.14) and for patients with LTBI (0.96, CI:0.82-1.13). Among 641 patients with drug-susceptible TB, 95% (333/352) of telehealth patients completed treatment within one year compared to 88% (254/289) of in-clinic patients (p = 0.002). This result is limited to the descriptive summary of this study population. During the COVID-19 pandemic, NYC Health Department provided telehealth to many patients with TB and LTBI of diverse demographics, and telehealth services were mostly comparable to in-clinic services.

## Author summary

During the COVID-19 pandemic, New York City Health Department tuberculosis (TB) clinics suspended non-essential in-person services, closed three of four clinics and rapidly shifted many services to telehealth. We wanted to understand the reach and effectiveness of this telehealth program. We compared patients with TB and latent TB infection who used telehealth with those who received only in-person care between April 2020 and December 2022. We found that patients with TB who used telehealth were similar in age and primary language to those seen in person. Visit completion was lower for telehealth early in the pandemic but became comparable to in-person visits over time. Among patients with drug-susceptible TB, a higher proportion of telehealth users completed treatment within one year compared to in-clinic patients. These findings suggest that telehealth was able to maintain continuity of TB care during a public health emergency and, in many cases, served as an acceptable alternative to in-person visits.

## Introduction

In recent years, use of telehealth has rapidly increased in healthcare delivery [1–4]. Telehealth is the use of telecommunications and information technology to remotely provide patient care services including health assessments and medical supervision and consultation [5].

In 2015, the World Health Organization (WHO) developed the End TB Strategy and established its Global Task Force on Digital Health for tuberculosis (TB) to support the development of digital health innovations for TB care and prevention [6,7]. The End TB Strategy recommends incorporating proven digital health tools with public health monitoring and evaluation of TB [6].

A systematic review with 89 randomized and observational studies demonstrated that telehealth services may be cost effective for patients and clinics, acceptable to users, and improve patient adherence to treatment [8]. A separate systematic review on adherence interventions with 129 randomized and observational studies found that electronic reminders and video observed therapy could improve TB treatment outcomes [9]. Additionally, telehealth may reduce the number of missed TB follow-up appointments among pediatric patients [10].

The use of remote patient monitoring and mobile health applications were essential for healthcare delivery during the COVID-19 pandemic [11–13], but were not without challenges. A 2020 mixed-methods survey of clinicians and clinic managers from geriatric medicine, internal medicine, and psychiatry practices reported concern that rapid increases in telehealth utilization exacerbated access to care disparities for older adults, those with limited internet access, and those needing interpretation [14]. In New York City (NYC), previous studies have reported disparities by race/ethnicity, age, and neighborhood with telehealth use [15–17]. Software and hardware issues were the main challenges for digital delivery of healthcare services [8]. However, with increased engagement of telehealth over time, patient willingness to use telehealth may increase within most demographic subgroups [18]. Furthermore, patients may be more likely to complete telehealth visits compared to in-person visits, in particular patients living in urban settings, or who required frequent medical visits and/or those living with chronic diseases [19]. Additionally, electronic directly observed therapy (DOT) in NYC has shown to have high adherence for TB treatment [20], but data linking telehealth visits with TB treatment completion is limited to date [8,21].

New York City Department of Health and Mental Hygiene (NYC Health Department) had four TB clinics that provided free TB medical services [22]. NYC Health Department has previously incorporated digital health into TB treatment services with follow-up care phone calls from nurses to increase treatment completion for latent TB infection (LTBI) [23] and by using asynchronous- and synchronous- DOT for patients with TB [20].

In late March 2020, when COVID-19 cases dramatically increased in NYC, three of four Health Department TB clinics suspended all services [24]. At that same time, the NYC Health Department TB staff leveraged its experience with digital health technologies to immediately offer telehealth to patients from either a central office or TB clinics. Patients with TB disease need continuity of care to minimize transmission to others, and to avoid developing drug-resistant TB [22]. Although telehealth services were offered to most patients at NYC Health Department TB clinics, patients who used telehealth, telehealth visit completion, and completion of TB treatment with use of telehealth may be different compared to traditional in-clinic services. The goal of this study is to assess the rapid utilization of telehealth and to compare telehealth services to in-clinic services for patients treated by NYC Health Department TB clinics during the COVID-19 pandemic.

## Materials and methods

### Objective and study design

Between April 2020 and December 2022, we conducted a prospective cohort study at NYC Health Department TB clinics. Patients with active TB or LTBI who received treatment at NYC Health Department TB clinics were included in this analysis. Patients who completed at least one telehealth visit during the study period were considered “telehealth patients.” A telehealth or in-clinic visit was considered complete if a patient attended their scheduled visit with a physician or nurse (herein referred to as providers).

We examined the frequency of telehealth visits during the COVID-19 pandemic in NYC; assessed differences between telehealth patients and in-clinic patients based on demographic, clinical, and socioeconomic characteristics; described operational and technological challenges to telehealth utilization; quantified the adjusted odds of visit completion by telehealth versus (vs) in-clinic visits; and reported TB treatment completion within one year for telehealth and in-clinic patients. These analyses aimed to identify any differences in the characteristics of patients using telehealth, and to determine whether the outcomes of visit and treatment completion telehealth patients were similar to those of in-clinic patients.

### Telehealth implementation

From April-June 2020, physicians conducted chart reviews of existing NYC Health Department TB clinic patients and transitioned eligible patients (e.g., did not require immediate in-person care and were able to use an electronic device) to telehealth services. In-person clinic appointments with a provider were reserved for patients with confirmed or probable TB or newly identified contacts to persons with infectious TB [25]. During this period, nearly all patients were given the option to have telehealth included as part of their treatment plan, so in-clinic-only patients were largely those who preferred to visit the clinic, did not feel comfortable operating an electronic device, or did not have access to an electronic device. No patient exclusions were made based on age or primary language spoken. Providers asked patients for their preferred telehealth visit type (telehealth-audio or telehealth-video). Patients with telehealth visits who required prescription refills had TB medications hand-delivered by a healthcare worker or by certified mail.

In July and August 2020, two closed clinics reopened for TB services with limited hours and staff updated telehealth documentation procedures based on feedback from patients and providers as protocols evolved. Patients returned to having initial in-person visits with a physician who determined if telehealth would be possible for the patient or if continued in-person care was necessary. Telehealth visits supplemented in-clinic visits, and patients continued to utilize in-clinic services. In January 2021, NYC Health Department TB clinics updated protocols with additional software options for telehealth-video and staff were retrained.

Providers called patients at their scheduled telehealth appointment times, reviewed TB treatment progress, inquired about patient reported side effects, and updated medication prescriptions. At the end of telehealth visits, providers determined whether the next visit should be via telehealth or in-person. Telehealth visits were conducted with Google Meet (Google, Mountain View, CA, USA) or FaceTime (Apple, Cupertino, CA, USA). These platforms were approved for telehealth use and compliant with the Health Insurance Portability and Accountability Act (HIPAA) compliant during the COVID-19 emergency period [26].

### Data source

Data were obtained from the NYC Health Department TB clinic electronic medical record (EMR) system and included all clinic patients with TB or LTBI who had at least one telehealth or in-person visit with a provider between April 2020 and December 2022. TB treatment completion data were collected from the NYC Health Department TB electronic disease surveillance and case management system (Maven 6.3.2., Conduent Inc, Florham Park, NJ), and these data included all individuals with active TB who lived in NYC and were diagnosed between April 2020 and December 2022.

### Outcomes and statistical analysis

Patient telehealth utilization was categorized as having attended at least one scheduled telehealth visit or completing no telehealth visit. A visit was complete if a patient attended their scheduled visit with a provider (by in-person or telehealth), else the visit was considered incomplete. A challenge to telehealth-video was defined as a documented reason by providers for switching from a scheduled telehealth-video visit to a telehealth-audio visit. Further, TB treatment completion was defined as completing standard TB treatment within one year for drug susceptible TB disease and assessed for those who started TB treatment. Patients who died, moved out of the United States, or had multi-drug resistant TB were excluded from this analysis.

Trends in telehealth usage were represented with a rolling 7-day average of visits from 2020 through 2022. We compared demographic, clinical characteristics, and treatment outcomes of telehealth and in-clinic patients, stratifying patients by active TB and LTBI. Bivariate analysis (Fischer’s exact tests for categorical variables and Wilcoxon ranked sum tests for continuous variables) compared telehealth patients and in-clinic patients with demographic and clinical characteristics with a significance threshold of p = 0.05. Multivariable logistic regression models were constructed using the demographic and clinical characteristics for patients with TB or LTBI to compare telehealth and in-clinic patients. Mixed (random and fixed) effects logistic regression models for visit completion were determined through sequential removal of explanatory variables based on the Bayesian information criterion and through consultation with subject matter experts. Clinic site and individual patients represented crossed random effects, whereas visit type (in-person vs telehealth), age, patient’s primary language, and whether or not visits occurred between April and June 2020 were fixed effects. Additional mixed effects logistic regression models compared telehealth with in-clinic visit completion in April and June 2020, when most TB clinics were closed, and separately in July 2020 to December 2022, when two TB clinics reopened. Among telehealth-video visits, a study coordinator conducted qualitative chart reviews to document challenges preventing visit completion. Lastly, the proportion of patients who completed TB treatment was compared between telehealth and in-clinic patients. RStudio (Version 4.2.3) [27] was used for data analysis and visualization.

### Ethics statement

The New York City Department of Health and Mental Hygiene Institutional Review Board categorized the project (Protocol #20–077) as exempt pursuant to 45 CFR §46.104(d)(4)(iii). This project only involved the use of existing data that is routinely collected for program services. Due to existing data protection provisions, consent was not obtained for this secondary research analysis as the research activities only involved information that is regulated for health care operations and public health purposes. This work was supported by New York City Department of Health and Mental Hygiene Bureau of Tuberculosis Control program funds.

## Results

**Fig 1:**
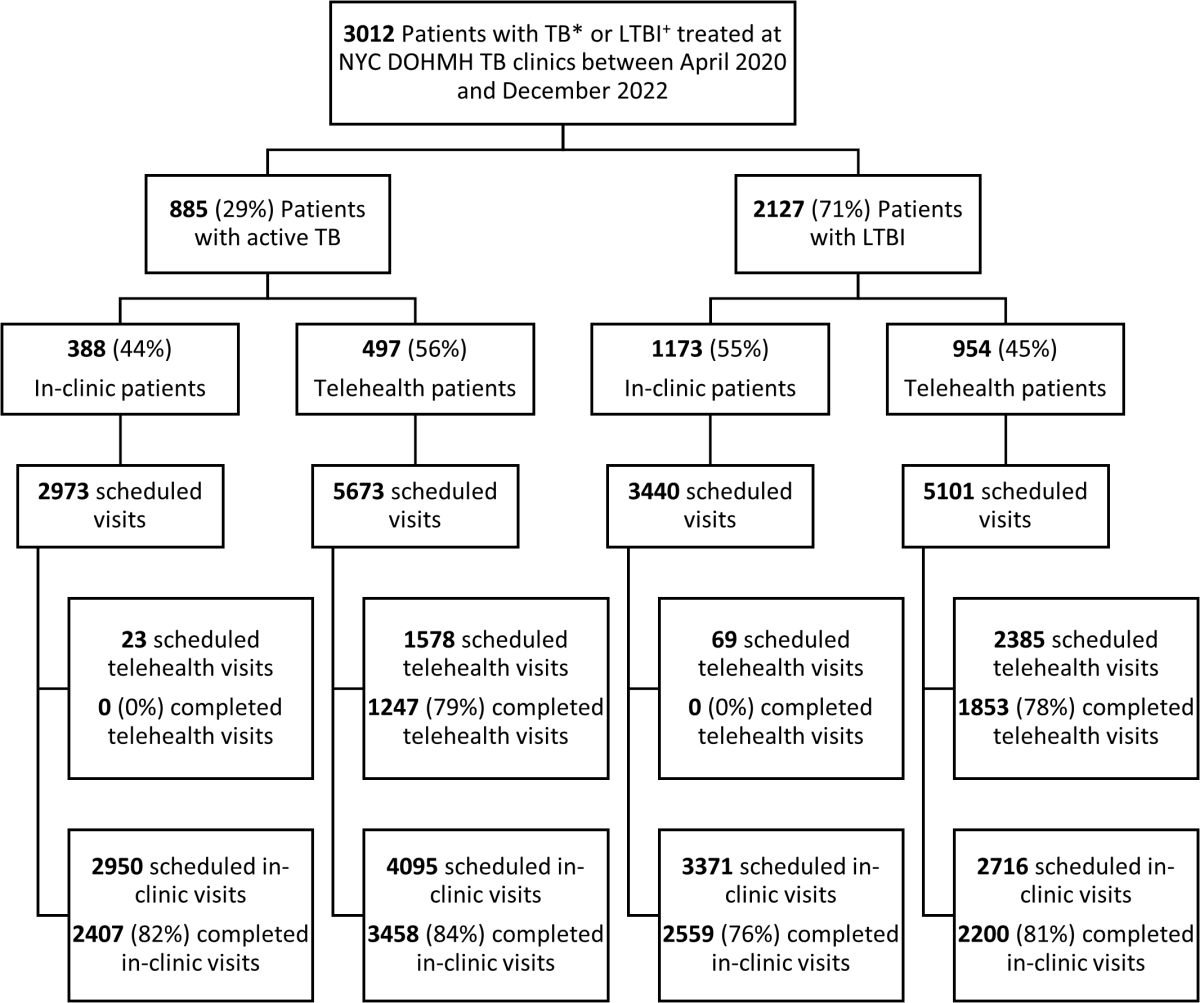
Flowchart of patients using NYC Health Department TB clinics telehealth services, April 2020 to December 2022. ^*^TB: Tuberculosis ^+^LTBI: Latent tuberculosis infection. ‡ Telehealth patients: Patient telehealth utilization was categorized as having attended at least one scheduled telehealth visit or completing no telehealth visit.

Between April 2020 and December 2022, 3012 patients diagnosed with TB or LTBI visited NYC Health Department TB clinics, of whom 885 (29%) had active TB and 2127 (71%) had LTBI (Fig 1). Of 3012 patients, 1451 (48%) completed at least one telehealth visit. Many telehealth patients (873/1451, 60%) had more than one telehealth visit. Telehealth patients with TB (n = 497) had 10 median visits per patient (IQR: 7, 12) with 2 median telehealth visits per patient (IQR: 1, 4). Telehealth patients with LTBI (n = 954) had 4 median visits per patient (IQR: 3, 5) with 2 median telehealth visits per patients (IQR: 1, 3). After July 2020, 61% (602/984) telehealth patients had in-clinic visits after their first telehealth visit.

**Fig 2:**
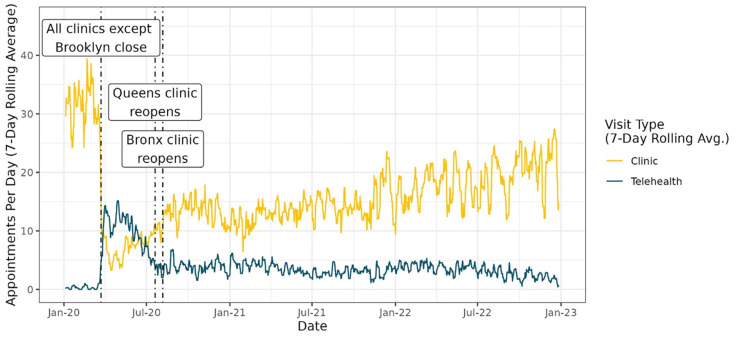
Rolling 7-day average of completed in-clinic and telehealth visits by day, 2020-2022.

After three of four TB clinics closed, daily in-clinic visits decreased and telehealth visits increased (Fig 2). Between April-June 2020, an average of 15 telehealth visits and an average of 10 in-clinic visits occurred per day. After two clinics reopened in August 2020, in-clinic visits increased and telehealth visits decreased. In August 2020, an average of 5 telehealth visits and 18 in-clinic visits per day occurred. In-clinic appointments continued to increase through the end of 2022.

### Demographic and clinical characteristics of TB clinic patients

Among patients with TB (n = 885), there was a greater proportion of female patients [41% (206/497) vs 29% (113/388), p < 0.001], patients with stable housing [98% (486/497) vs 94% (363/388), p < 0.001], and patients with only extrapulmonary disease [15% (75/487) vs 9% (33/388), p = 0.004] among telehealth patients than in-clinic patients (Table 1). Telehealth utilization was significantly different by patients’ primary TB clinics (i.e., the clinic they attended the greatest number of times during the study period) (p < 0.001 Table 1). Among patients whose primary clinic was in the Bronx, 68% (145/214) utilized telehealth. No other significant associations were observed between telehealth utilization and age (p = 0.31), primary language (p = 0.37), or other characteristics assessed (Table 1).

**Table 1:** Demographic and clinical characteristics of patients with TB and LTBI by telehealth use at NYC Health Department TB clinics between 4/1/2020 and 12/31/2022.

	Patients with TB					Patients with latent TB infection (LTBI)				
	Telehealth Patients^*^ (n = 497)	%	In-clinic Patients (n = 388)	%	p-value^†^	Telehealth Patients (n = 954)	%	In-clinic Patients (n = 1173)	%	p-value
**Age (Median [Interquartile range])**	47 [33, 64]		51 [34, 65]		0.31	41 [26, 55]		45 [32, 57]		**<0.001**
**Female sex**	206	41%	113	29%	**<0.001**	493	52%	554	47%	**0.04**
**Primary language**					0.37					**<0.001**
**Spanish**	144	29%	108	28%		387	41%	393	34%	
**English**	134	27%	89	23%		269	28%	409	35%	
**Chinese**	81	16%	76	20%		60	6%	58	5%	
**Other**	138	28%	115	30%		238	25%	313	27%	
**Non U.S.-Born**	435	88%	345	89%	0.60	827	87%	952	81%	**<0.001**
**Stably housed**	486	98%	363	94%	**<0.001**	744	78%	866	74%	**<0.001**
**Area-based poverty level** ^‡^					0.42					0.06
**Low (<10%)**	82	16%	76	20%		174	18%	232	20%	
**Medium (10 to <20%)**	206	41%	167	43%		411	43%	520	44%	
**High (20 to <30%)**	102	21%	80	21%		178	19%	238	20%	
**Very high (30–100%)**	99	20%	61	16%		173	18%	157	13%	
**Unknown**	8	2%	4	1%		18	2%	26	2%	
**Primary clinic** ^§^					**<0.001**					**<0.001**
**Bronx**	145	29%	69	18%		212	22%	208	18%	
**Brooklyn**	192	39%	160	41%		418	44%	577	49%	
**Queens**	160	32%	159	41%		292	31%	388	33%	
**Other/None**	0	0%	0	0%		32	3%	0	0%	
**Multidrug-resistant TB**	8	2%	9	2%	0.47	N/A		N/A		
**Disease site: Extrapulmonary only**	75	15%	33	9%	**0.004**	N/A		N/A		
**Sputum smear positive**	285	57%	217	56%	0.68	N/A		N/A		

* Those who successfully completed at least one telehealth visit during the duration of their care were considered “telehealth patients”. Patients who only had in-person clinic visits were considered “in-clinic patients”. Demographic information and TB classification were based on patient’s last visit status during the study period.

† Two-tailed Fisher exact for proportions and Wilcoxon rank sum tests for medians were used to determine statistical differences between patients who used telehealth and those who did not, with a significance threshold of p = 0.05.

‡ Based on 2017–2021 American Community Survey data on the proportion of ZIP code residents living below the federal poverty level [28].

§ Each patient’s primary clinic was defined as the clinic that they attended the greatest number of times during the study period.

Among patients with LTBI (n = 2127), telehealth patients had a lower median age (41 vs 45, p < 0.001), and were more likely to be female [52% (493/954) vs 47% (554/1173), p = 0.04], be stably housed [78% (744/954) vs 74% (866/1173), p < 0.001)], and be born outside the US [87% (827/954) vs 81% (952/1173), p < 0.001]. There were also significant differences in the proportion of patients who used telehealth based on primary language (p < 0.001) and primary clinic attended (p < 0.001) (Table 1). Specifically, among patients whose primary language was Spanish, 50% (387/780) used telehealth and among patients whose primary clinic was in the Bronx, 50% (212/420) used telehealth (Table 1).

Each additional year of age for patients with LTBI was associated with a 1% decrease in telehealth usage (adjusted odds ratio (AOR): 0.99, confidence interval (CI): 0.98-0.99, p < 0.001) compared to using in-clinic visits, while adjusting for variables including patient sex, housing status and primary language (S1 Table). However, there was no significant association between age and telehealth usage among patients with TB (AOR: 1, CI: 0.99 – 1.01, p = 0.78), compared to using in-clinic visits, while adjusting for the same variables (S1 Table). Additional sensitivity analyses were done (splitting the dataset with the April-June 2020 period and the subsequent July 2020-December 2022 periods due to changing telehealth protocols) and yielded concurrent results (S2 and S3 Tables).

### Challenges to telehealth-video

Telehealth was primarily conducted by audio from April through December 2020. After telehealth-video protocols were updated, patients scheduled 2660 telehealth visits between January 2021 and December 2022, 730 (27%) telehealth-video visits and 1934 (73%) telehealth-audio visits. Telehealth-video visit proportion among all telehealth visits increased from 4% (5/138) in January 2021 to 40% (38/95) in September 2022 (S1 Fig).

Of 730 scheduled telehealth-video visits, 13% (93/730) were incomplete, 75% (545/730) completed as telehealth-video visits, and 13% (92/730) became telehealth-audio visits. Of 1934 scheduled telehealth-audio visits, 11% (212/1934) were incomplete and 89% (1722/1934) completed.

Of 92 visits that switched from telehealth-video to telehealth-audio, 60% (55/92) had documented challenges by the physician. Documented challenges included: patient or provider reported technical difficulties [76% (42/55)], patient request during their visit [13%, (7/55)], patient’s background preventing use of video [7%, (4/55)], and difficulties with translation services [4%, (2/55)].

After January 2021, patients continued to use telehealth-audio more than telehealth-video (1830 vs 545 completed visits). However, the overall proportion of telehealth visits decreased from 53% (1266/2053) in April to June 2020 to 9% (234/2640) in October to December 2022.

### Telehealth visit completion

**Fig 3:**
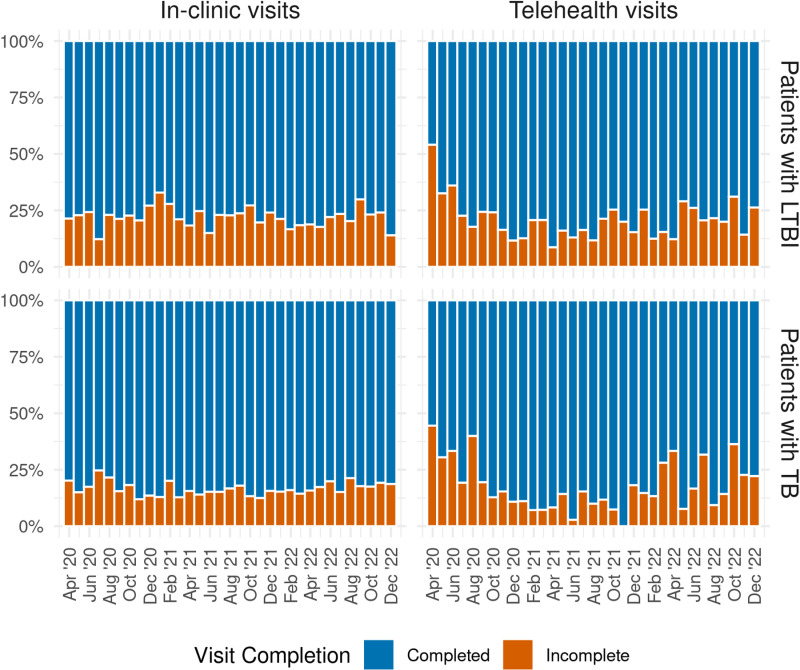
Monthly proportion of complete and incomplete visits of in-clinic and telehealth visits for patients with TB* and LTBI†, April 2020 to December 2022. *TB: Tuberculosis †LTBI: Latent tuberculosis infection.

Patients with TB (n = 885) completed 78% (1247/1601) of their scheduled telehealth visits compared to 83% (5865/7045) of scheduled in-clinic visits (p < 0.001, Fig 3).

For patients with TB, a higher proportion of all visits was completed by female patients (84% vs 81%, p < 0.001), patients born outside of the US (83% vs 76%, p < 0.001), and patients whose primary language was Chinese vs English (87% vs 79%, p < 0.001).

Patients with LTBI (n = 2127) completed 76% (1853/2454) of telehealth visits compared to 78% (4759/6087) of in-clinic visits (p = 0.01). A higher proportion of all visits was completed by patients over 45 vs 45 and under (80% vs 75%, p < 0.001), by patients born outside of the US (78% vs 74%, p = 0.004), and patients whose primary language was Chinese vs English (86% vs 76%, p < 0.001).

Overall, patients with TB had a 23% reduction in adjusted odds (AOR: 0.77; CI: 0.65-0.91) of completing telehealth visits in comparison to in-clinic visits when controlling for patient, clinic, age, primary language, and visits occurring between April-June 2020 or between July 2020 and December 2022 (Table 2). Patients with LTBI also had a 12% reduction in adjusted odds (AOR: 0.88; CI: 0.76-1.03) of completing a telehealth visit when controlling for the same characteristics, however this finding was not statistically significant.

**Table 2:** Mixed effects logistic regression model estimates for completing a visit for patients with TB^*^ or LTBI^†^, April 2020 to December 2022.

	Patients with TB			Patients with LTBI		
	Visit completion			Visit completion		
*Predictors*	*AOR*	*CI*	*p-value*	*AOR*	*CI*	*p-value*
Patient age^‡^	1.01	1.00 - 1.01	**0.026**	1.01	1.00 - 1.01	**0.004**
In-clinic visit	*Reference*			*Reference*		
Telehealth visit	0.77	0.65 - 0.92	**0.003**	0.88	0.76 - 1.03	0.120
July 2020 - December 2022 visit	*Reference*			*Reference*		
April - June 2020 visit	0.60	0.48 - 0.75	**<0.001**	0.42	0.34 - 0.51	**<0.001**
English^§^	*Reference*			*Reference*		
Spanish	1.02	0.80 - 1.30	0.866	0.99	0.85 - 1.15	0.902
Chinese	1.54	1.14 - 2.10	**0.005**	1.71	1.25 - 2.34	**0.001**
Other	1.43	1.11 - 1.84	**0.005**	1.27	1.08 - 1.51	**0.005**

* TB: Tuberculosis

† LTBI: Latent tuberculosis infection

‡ Patient age, in years, at time of visit.

§ Language refers to primary language spoken by the patient. “Other” refers to the 82 languages spoken by patients, including Bengali, French, Nepali, Tibetan, and Tagalog, among others

AOR: Adjusted odds ratio.

CI: Confidence interval

Individual patients (Patients with TB: N = 885; Patients with LTBI: N = 2127) and each clinic were included as random effects in the models.

Additional analyses were done (splitting the dataset with the April-June 2020 period and the subsequent July 2020-December 2022 period) where results differed. Between April-June 2020, there were significantly reduced adjusted odds of completing a telehealth visit compared to an in-clinic visit for patients with TB (AOR: 0.15; CI: 0.06-0.37), and for patients with LTBI (AOR: 0.41; CI: 0.29-0.58) while controlling for patient, clinic, age, and primary language (S4 Table). For the July 2020-December 2022 period, the adjusted odds of completing a telehealth visit compared to an in-clinic visit were not statistically different for patients with TB (AOR: 0.94; CI 0.77-1.14) and for patients with LTBI (AOR: 0.96; CI: 0.82-1.13) while controlling for patient, clinic, age, and primary language (S5 Table).

### Treatment completion

Between April 2020 and December 2022, of 641 patients eligible to complete TB treatment within one year, 587 (92%) completed their TB treatment course. Among telehealth patients with TB, 29% (145/497) were excluded from the TB treatment completion analysis, 124 (86%) had TB diagnosed outside of April 2020 to December 2022 and 21 (14%) were ineligible to complete TB treatment within one year. Among in-clinic patients with TB, 26% (99/388) were excluded, 61 (62%) had TB diagnosed outside of April 2020 to December 2022, and 38 (38%) were ineligible to complete TB treatment within one year.

Of 641 patients with TB, 95% (333/352) of telehealth patients completed treatment within one year compared to 88% (254/289) of in-clinic patients (p = 0.002). Telehealth patients who completed treatment had more median visits with providers than in-clinic patients who completed treatment [11 (IQR: 9–13) vs 8 (IQR: 6–10), p < 0.001]. However, there was no difference in the number of missed appointments between telehealth and in-clinic patients who completed treatment (p = 0.3).

Of the 19 telehealth patients who did not complete treatment within one year, 74% (14/19) completed treatment after one year, 16% (3/19) were lost to follow-up, and 11% (2/19) refused TB treatment and did not return to the clinic. Of the 35 in-clinic patients who did not complete treatment within one year, 51% (18/35) completed treatment after one year, 31% (11/35) were lost, and 17% (6/35) refused TB treatment and did not return.

## Discussion

Throughout the COVID-19 pandemic, over 450 patients with TB and over 900 patients with LTBI used telehealth services at NYC Health Department TB clinics. The use of telehealth increased between April-June 2020 and decreased after TB clinics reopened. Telehealth visit completion was comparable to in-clinic visit completion for most of the study period except during the height of the COVID-19 pandemic in NYC (April-June 2020). The difference in treatment completion rates (95% versus 88%) may have been due to differences in patient characteristics – we conclude simply that TB treatment completion within one year among telehealth patients and in-clinic patients was high. Telehealth was an essential part of TB care through NYC Health Department TB clinics.

In our study population, patients with TB accessed telehealth at similar proportions across age and primary language spoken. Similarly, other studies reported that as utilization of telehealth increased, disparities in telehealth usage between race and age groups decreased [29,30]. In contrast, other studies reported telehealth access can be limited for individuals who are older or whose primary language is not English [14,31]. In our study population, older patients with LTBI used telehealth less, which is similar to previous reports [16,31]. Yet, telehealth usage among patients with LTBI whose primary language was Spanish was high, which may be due to the availability of Spanish-speaking providers, translation services, and some patients’ English-language proficiency in addition to Spanish. Other studies report telehealth usage was less common among Hispanic patients or patients needing interpretation services [14,32].

The preference for telehealth audio visits among our study population was similar to two findings where respondents preferred phone visits over video visits [33,34]. Although the NYC Health Department TB clinics continues to offer telehealth as a part of standard of care, our study population and clinical providers showed a decline in both preference and utilization of telehealth by December 2022. Further analysis is needed to explore this trend. This is consistent with a nationwide trend reported by the 2023 Trilliant Health report which showed a 45.8% decline in telehealth visits from 76.7 million visits in April-June 2020 to 41.5 million visits in October to December 2022 [35].

Previous studies reported higher telehealth visit completion than in-clinic visit completion in community-based settings [19,36]. Low telehealth visit completion in April-June 2020 may have been due to the rapid transition to telehealth services during which patients were quickly informed of visits converting to telehealth. As program practices were updated and staff received trainings on telehealth software, telehealth visit completion rates improved. Excluding the months of April-June 2020, and for the majority of the study period, visit completion did not statistically differ between telehealth and in-clinic, which reflects previous studies comparing efficacy between in-person and digital health interventions in this population [20].

TB treatment completion within one year was higher for both telehealth patients and in-clinic patients in comparison to national averages from 2021 reported by the Centers for Disease Control [37]. While it is encouraging that telehealth patients experienced higher TB treatment completion, we limit our interpretation of this result to the descriptive summary of this study population. Telehealth patients with TB who completed treatment within one year had more overall visits in comparison to in-clinic patients, potentially indicating a more engaged population. However, there may also be intrinsic factors of telehealth such as convenience of remote access, technological literacy, or the nature of the patient-provider interaction in a virtual setting, that could have influenced treatment completion results. We also lacked the ability to measure the morbidity of TB disease experienced by each patient which impacts their ability to complete treatment.

### Limitations

In our study population, patient or provider perceptions of telehealth compared to in-clinic visits were not assessed. A recent multi-site cohort analysis of patient satisfaction for telehealth vs in-clinic care indicated that there was no difference in patient satisfaction during the COVID-19 pandemic [38], but levels of patient satisfaction could be different in our study population. Future work could incorporate patient reported perceptions, technological barriers, and outcomes to determine satisfaction with telehealth services in Health Department TB clinics. Additionally, no data was collected on the cost to the city of providing services via telehealth versus in-person (services are provided free of cost to patients).

The use of telehealth varied over time and by clinic. Changes in the severity of the pandemic during the study period could have confounded some analyses. While the experiences at the clinic that remained partially open during the peak of the emergency in NYC was different from those that closed temporarily, our findings cannot be generalized broadly, as all clinics studied were among the TB clinics run by the NYC Health Department. Some telehealth patients used telehealth frequently, some patients had only one telehealth visit, and many patients had both telehealth and in-clinic services during their care. TB clinics and staff were operating on a limited schedule; staff availability and appointment availability were limited during the study period due to the COVID-19 pandemic, clinic closures, and staffing shortages. These program operations impacted the implementation and evaluation of telehealth in the clinics.

Due to the nature of this observational study, there are additional limitations. It was not possible to randomize patients, blind patients, or blind staff to the intervention in this public health setting, which resulted in differences between the cohorts of telehealth patients and in-clinic patients, many of which were controlled for in our analyses. Patient-level variables associated with the choice to participate in telehealth could confound analyses of patient outcomes. Since data were collected in the EMR for routine public health purposes, this both limited the set of research questions that could be addressed and could have biased study outcomes. Finally, the lack of long-term follow up means that it is not yet possible for to know which of the patients included in this study will eventually have relapses of TB disease.

## Conclusion

During the COVID-19 pandemic, in a large urban public health setting, NYC Health Department TB clinics rapidly provided telehealth services to patients with TB and LTBI of diverse demographic characteristics to support visit completion and TB treatment completion. NYC Health Department telehealth services were comparable to traditional in-clinic services.

## Supporting information

S1 Table Multivariable logistic regression model estimates for whether patients used telehealth. The sample includes patients at NYC Health Department TB clinics between April 2020 and December 2022. Adjusted odds ratios are calculated for selected demographic and clinical characteristics of patients with active TB disease or latent TB infection. Those who successfully completed at least one telehealth visit during the duration of their care were considered “telehealth patients”. Patients who only had in-person clinic visits were considered “in-clinic patients”. Demographic information and TB classification were based on patient’s last visit status during the study period. Area-based poverty level uses the 2017–2021 American Community Survey data on the proportion of ZIP code residents living below the federal poverty level. The Variance Inflation Factor was between 1 and 1.7 for each variable, suggesting that multicollinearity was not problematic in this model.
(DOCX)

S2 Table Multivariable logistic regression model estimates for whether patients used telehealth (April to June 2020). The sample includes patients at NYC Health Department TB clinics between April 2020 and June 2020. Adjusted odds ratios are calculated for selected demographic and clinical characteristics of patients with active TB disease or latent TB infection. Those who successfully completed at least one telehealth visit during the duration of their care were considered “telehealth patients”. Patients who only had in-person clinic visits were considered “in-clinic patients”. Demographic information and TB classification were based on patient’s last visit status during the study period. Area-based poverty level uses the 2017–2021 American Community Survey data on the proportion of ZIP code residents living below the federal poverty level. Primary clinic cannot be analyzed for this period because only the Brooklyn clinic was open during this period; thus, no in-clinic-only patients exist for the other clinics during this period.
(DOCX)

S3 Table Multivariable logistic regression model estimates for whether patients used telehealth (July 2020 to December 2022). The sample includes patients at NYC Health Department TB clinics between July 2020 and December 2022. Adjusted odds ratios are calculated for selected demographic and clinical characteristics of patients with active TB disease or latent TB infection. Those who successfully completed at least one telehealth visit during the duration of their care were considered “telehealth patients”. Patients who only had in-person clinic visits were considered “in-clinic patients”. Demographic information and TB classification were based on patient’s last visit status during the study period. Area-based poverty level uses the 2017–2021 American Community Survey data on the proportion of ZIP code residents living below the federal poverty level.
(DOCX)

S4 Table Mixed effects logistic regression model estimates for completing a visit for patients with TB* or LTBI†, for the height of pandemic: April 2020 through June 2020. ^*^ TB: Tuberculosis. ^†^ LTBI: Latent tuberculosis infection. ^‡^ Patient age, in years, at time of visit. ^§^ Language refers to primary language spoken by the patient. “Other” refers to the 82 languages spoken by patients, including Bengali, French, Nepali, Tibetan, and Tagalog, among others. AOR: Adjusted odds ratio, CI: Confidence interval.
(DOCX)

S5 Table Mixed effects logistic regression model estimates for completing a visit for patients with TB* or LTBI†, July 2020 to December 2022. ^*^ TB: Tuberculosis. ^†^ LTBI: Latent tuberculosis infection. ^‡^ Patient age, in years, at time of visit. ^§^ Language refers to primary language spoken by the patient. “Other” refers to the 82 languages spoken by patients, including Bengali, French, Nepali, Tibetan, and Tagalog, among others. AOR: Adjusted odds ratio, CI: Confidence interval.
(DOCX)

S1 Fig Monthly proportion of telehealth-video visits scheduled among all telehealth visits at NYC Health Department TB Clinics, April 2020 to December 2022. (TIF)
