# Supplementary material for: An evaluation of telehealth services at New York City tuberculosis clinics throughout the COVID-19 pandemic
Source: PLOS Digit Health. 2025 Jun 24;4(6):e0000898. doi: 10.1371/journal.pdig.0000898 (PMC12186896; doi:10.1371/journal.pdig.0000898)
Supplement: S2 Table — The sample includes patients at NYC Health Department TB clinics between April 2020 and June 2020. Adjusted odds ratios are calculated for selected demographic and clinical characteristics of patients with active TB disease or latent TB infection. Those who successfully completed at least one telehealth visit during the duration of their care were considered “telehealth patients”. Patients who only had in-person clinic visits were considered “in-clinic patients”. Demographic information and TB classification were based on patient’s last visit status during the study period. Area-based poverty level uses the 2017–2021 American Community Survey data on the proportion of ZIP code residents living below the federal poverty level. Primary clinic cannot be analyzed for this period because only the Brooklyn clinic was open during this period; thus, no in-clinic-only patients exist for the other clinics during this period. (DOCX) [file pdig.0000898.s002.docx]

**S2 Table. Multivariable logistic regression model estimates for whether patients used telehealth (April to June 2020).**

|  | Active TB | | Latent TB Infection | |
| --- | --- | --- | --- | --- |
|  | *Adjusted odds ratios telehealth vs in-clinic (95% CI)* | *p-value (adjusted model)* | *Adjusted odds ratios telehealth vs in-clinic (95% CI)* | *p-value (adjusted model)* |
| **Age** | 1.01 (0.99 – 1.02) | 0.56 | 0.98 (0.96 – 0.99) | **0.002** |
| **Region born** |  |  |  |  |
| **US** | Reference | Reference | Reference | Reference |
| **Non-US** | 1.33 (0.43 –3.88) | 0.61 | 2.11 (1.02 –4.36) | 0.04 |
| **Sex** |  |  |  |  |
| **Female** | Reference | Reference | Reference | Reference |
| **Male** | 0.56 (0.27 –1.12) | 0.11 | 0.64 (0.38 – 1.06) | 0.08 |
| **Housing status** | |  |  |  |
| **Not stably housed** | Reference | Reference | Reference | Reference |
| **Stably housed** | 14.32 (1.84–300.24) | **0.02** | 3.47 (1.57 – 7.84) | **0.002** |
| **Primary language** | |  |  |  |
| **English** | Reference | Reference | Reference | Reference |
| **Spanish** | 1.23 (0.46 – 3.28) | 0.67 | 2.42 (1.22– 4.88) | **0.01** |
| **Chinese** | 0.91 (0.28 –2.94) | 0.87 | 1.21 (0.40 –3.94) | 0.74 |
| **Other** | 0.70 (0.25 – 1.90) | 0.49 | 1.04 (0.51 –2.15) | 0.91 |
| **Area-based poverty level** | |  |  |  |
| **Low** | 1.4 (0.45 – 4.78) | 0.57 | 2.25 (1.03– 5.05) | **0.05** |
| **Medium** | 1.9 (0.80 – 4.55) | 0.15 | 2.31 (1.22–4.41) | **0.01** |
| **High** | Reference | Reference | Reference | Reference |
| **Very high** | 1.35 (0.51 –3.64) | 0.54 | 3.37 (1.60– 10.78) | **0.004** |
| **Unknown** | 0.37 (0.01 –11.10) | 0.61 | 0.70 (0.16 –3.15) | 0.63 |

The sample includes patients at NYC Health Department TB clinics between April 2020 and June 2020. Adjusted odds ratios are calculated for selected demographic and clinical characteristics of patients with active TB disease or latent TB infection. Those who successfully completed at least one telehealth visit during the duration of their care were considered “telehealth patients”.  Patients who only had in-person clinic visits were considered “in-clinic patients”. Demographic information and TB classification were based on patient’s last visit status during the study period. Area-based poverty level uses the 2017-2021 American Community Survey data on the proportion of ZIP code residents living below the federal poverty level.

Primary clinic cannot be analyzed for this period because only the Brooklyn clinic was open during this period; thus, no in-clinic-only patients exist for the other clinics during this period.
