# Supplementary material for: An evaluation of telehealth services at New York City tuberculosis clinics throughout the COVID-19 pandemic
Source: PLOS Digit Health. 2025 Jun 24;4(6):e0000898. doi: 10.1371/journal.pdig.0000898 (PMC12186896; doi:10.1371/journal.pdig.0000898)
Supplement: S4 Table — * TB: Tuberculosis. † LTBI: Latent tuberculosis infection. ‡ Patient age, in years, at time of visit. § Language refers to primary language spoken by the patient. “Other” refers to the 82 languages spoken by patients, including Bengali, French, Nepali, Tibetan, and Tagalog, among others. AOR: Adjusted odds ratio, CI: Confidence interval. (DOCX) [file pdig.0000898.s004.docx]

**S4 Table.** Mixed effects logistic regression model estimates for completing a visit for patients with TB* or LTBI†, for the height of pandemic: April 2020 through June 2020

|  | Patients with TB | | | Patients with LTBI | | |  |
| --- | --- | --- | --- | --- | --- | --- | --- |
|  | **Visit completion** | | | **Visit completion** | | |  |
| *Predictors* | *AOR* | *CI* | *p-value* | *AOR* | *CI* | *p-value* | |
| Patient age^‡^ | 1.01 | 1.00 - 1.02 | 0.229 | 1.00 | 0.99 - 1.01 | 0.532 | |
| In-clinic visit | *Reference* | |  | *Reference* | |  | |
| Telehealth visit | 0.15 | 0.06 - 0.37 | **<0.001** | 0.41 | 0.29 - 0.58 | **<0.001** | |
| English^§^ | *Reference* | |  | *Reference* | |  | |
| Spanish | 1.25 | 0.79 - 1.98 | 0.348 | 0.92 | 0.64 - 1.33 | 0.661 | |
| Chinese | 0.92 | 0.53 - 1.59 | 0.760 | 1.23 | 0.59 - 2.53 | 0.582 | |
| Other | 1.45 | 0.90 - 2.34 | 0.128 | 0.93 | 0.61 - 1.40 | 0.712 | |

^*^ TB: Tuberculosis

^†^ LTBI: Latent tuberculosis infection

^‡^ Patient age, in years, at time of visit.

^§^ Language refers to primary language spoken by the patient. “Other” refers to the 82 languages spoken by patients, including Bengali, French, Nepali, Tibetan, and Tagalog, among others

AOR: Adjusted odds ratio

CI: Confidence interval
