# Supplementary material for: An evaluation of telehealth services at New York City tuberculosis clinics throughout the COVID-19 pandemic
Source: PLOS Digit Health. 2025 Jun 24;4(6):e0000898. doi: 10.1371/journal.pdig.0000898 (PMC12186896; doi:10.1371/journal.pdig.0000898)
Supplement: S5 Table — * TB: Tuberculosis. † LTBI: Latent tuberculosis infection. ‡ Patient age, in years, at time of visit. § Language refers to primary language spoken by the patient. “Other” refers to the 82 languages spoken by patients, including Bengali, French, Nepali, Tibetan, and Tagalog, among others. AOR: Adjusted odds ratio, CI: Confidence interval. (DOCX) [file pdig.0000898.s005.docx]

**S5 Table**. Mixed effects logistic regression model estimates for completing a visit for patients with TB* or LTBI†, July 2020 to December 2022

|  | Patients with TB | | | Patients with LTBI | | |
| --- | --- | --- | --- | --- | --- | --- |
|  | **Visit completion** | | | **Visit completion** | | |
| *Predictors* | *AOR* | *CI* | *p-value* | *AOR* | *CI* | *p-value* |
| Patient age^‡^ | 1.00 | 1.00 - 1.01 | 0.106 | 1.01 | 1.00 - 1.01 | **0.005** |
| In-clinic visit | *Reference* | |  | *Reference* | |  |
| Telehealth visit | 0.94 | 0.77 - 1.14 | 0.534 | 0.96 | 0.81 - 1.13 | 0.634 |
| English^§^ | *Reference* | |  | *Reference* | |  |
| Spanish | 0.99 | 0.76 - 1.28 | 0.912 | 1.01 | 0.86 - 1.19 | 0.887 |
| Chinese | 1.89 | 1.35 - 2.65 | **<0.001** | 1.88 | 1.33 - 2.66 | **<0.001** |
| Other | 1.46 | 1.11 - 1.91 | **0.006** | 1.35 | 1.13 - 1.62 | **0.001** |

^*^ TB: Tuberculosis

^†^ LTBI: Latent tuberculosis infection

^‡^ Patient age, in years, at time of visit.

^§^ Language refers to primary language spoken by the patient. “Other” refers to the 82 languages spoken by patients, including Bengali, French, Nepali, Tibetan, and Tagalog, among others

AOR: Adjusted odds ratio

CI: Confidence interval
