# Supplementary figures and images for: An evaluation of telehealth services at New York City tuberculosis clinics throughout the COVID-19 pandemic
Source: PLOS Digit Health. 2025 Jun 24;4(6):e0000898. doi: 10.1371/journal.pdig.0000898 (PMC12186896; doi:10.1371/journal.pdig.0000898)

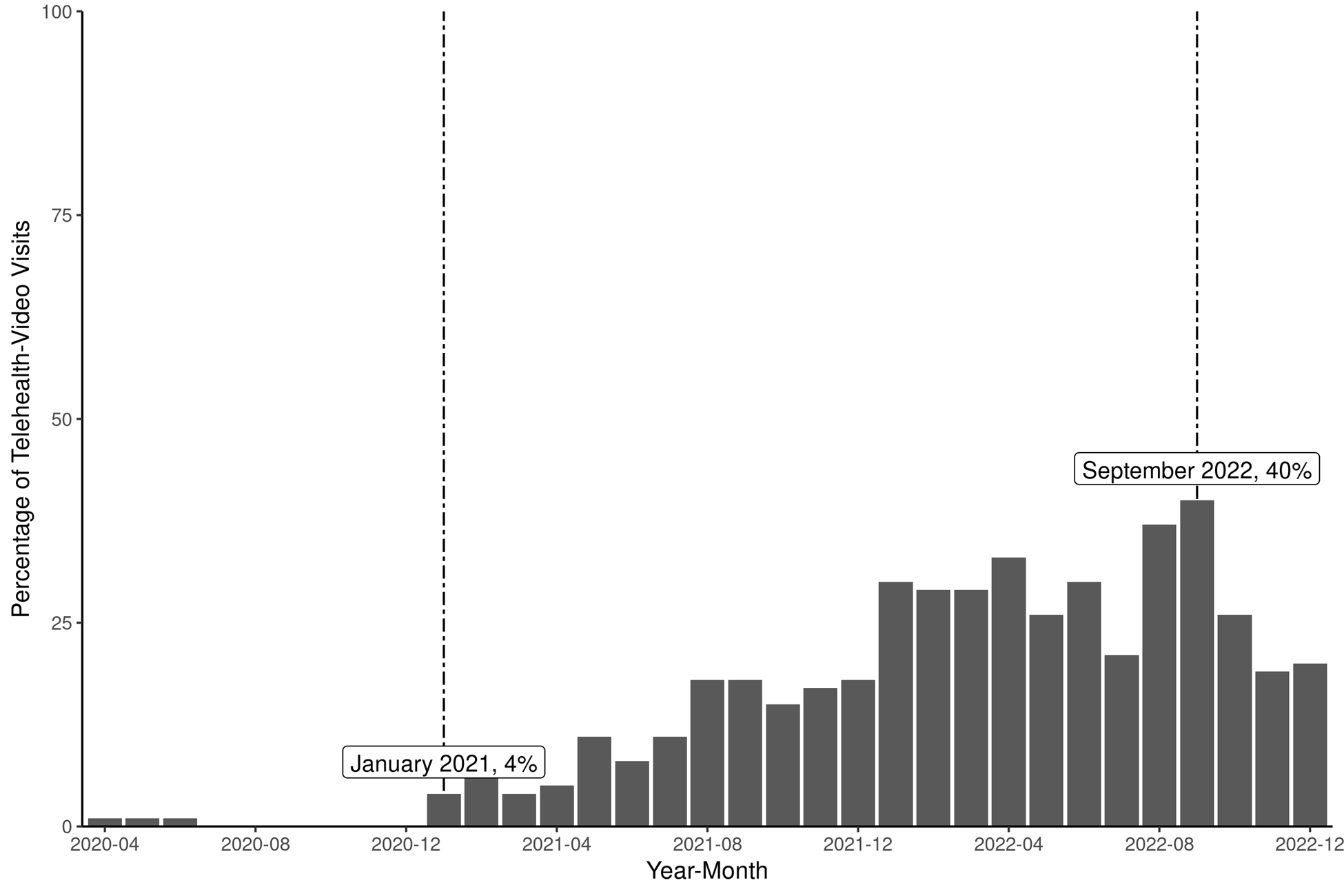

Supplement: S1 Fig — (TIF) [file pdig.0000898.s006.tif]
